# Preservation of protein clefts in comparative models

**DOI:** 10.1186/1472-6807-8-2

**Published:** 2008-01-16

**Authors:** David Piedra, Sergi Lois, Xavier de la Cruz

**Affiliations:** 1 Institut de Recerca Biomèdica, C/Josep Samitier, 1-5, 08028 Barcelona, Spain; 2 Institució Catalana de Recerca i Estudis Avançats (ICREA), Barcelona, Spain; 3 Instituto de Biología Molecular de Barcelona, CID, Consejo Superior de Investigaciones Científicas (CSIC), Barcelona, Spain

## Abstract

**Background:**

Comparative, or homology, modelling of protein structures is the most widely used prediction method when the target protein has homologues of known structure. Given that the quality of a model may vary greatly, several studies have been devoted to identifying the factors that influence modelling results. These studies usually consider the protein as a whole, and only a few provide a separate discussion of the behaviour of biologically relevant features of the protein. Given the value of the latter for many applications, here we extended previous work by analysing the preservation of native protein clefts in homology models. We chose to examine clefts because of their role in protein function/structure, as they are usually the locus of protein-protein interactions, host the enzymes' active site, or, in the case of protein domains, can also be the locus of domain-domain interactions that lead to the structure of the whole protein.

**Results:**

We studied how the largest cleft of a protein varies in comparative models. To this end, we analysed a set of 53507 homology models that cover the whole sequence identity range, with a special emphasis on medium and low similarities. More precisely we examined how cleft quality – measured using six complementary parameters related to both global shape and local atomic environment, depends on the sequence identity between target and template proteins. In addition to this general analysis, we also explored the impact of a number of factors on cleft quality, and found that the relationship between quality and sequence identity varies depending on cleft rank amongst the set of protein clefts (when ordered according to size), and number of aligned residues.

**Conclusion:**

We have examined cleft quality in homology models at a range of seq.id. levels. Our results provide a detailed view of how quality is affected by distinct parameters and thus may help the user of comparative modelling to determine the final quality and applicability of his/her cleft models. In addition, the large variability in model quality that we observed within each sequence bin, with good models present even at low sequence identities (between 20% and 30%), indicates that properly developed identification methods could be used to recover good cleft models in this sequence range.

## Background

In order to make full use of the growing amount of sequence information, in terms of increasing our knowledge of protein function, engineering new variants of known proteins, developing biomedical applications, etc, structural information is clearly required [1-6]. Indeed, one of the most important challenges in the post-genomics era is to fill the gap between the large number of known protein sequences and the still relatively small number of known structures [6-9]. Structural genomics projects have addressed this challenge and have led to the design and development of high-throughput production pipelines for structure determination [2,10-15]. This considerable research effort is starting to give results and recent reports show a clear increase in the number of known structures, and particularly of structures showing new folds, solved in structural genomics projects [16-20].

Providing experimental structures for all possible proteins clearly exceeds our present capacity. Therefore, the yield of structural genomics projects is increased by the use of comparative/homology modelling tools [1,2,6,9,10,13,16,21]. Indeed, the latter are of great importance as they allow the extension of the knowledge provided by structural genomics projects by at least one order of magnitude [6,7,22]. However, the usefulness of homology models varies and is determined by their quality [1,23,24]. Drug design (probably the most demanding application of homology models) requires high quality models that are usually obtained for sequence identity (seq.id.) levels above 70% between the target and template [23,24]. Useful designs of pseudo-molecules fitting the active site of an enzyme, which can be employed for screening small-compound databases, can be obtained at seq.id. of around 30% [25]; medium to high-accuracy models can be applied to interpret the damaging effect of point mutations [2,24], etc. A series of independent studies [24,26-29], as well as the results of CASP experiments [30-41], give the user of comparative modelling a good idea of the model's overall performance, and how the latter can be estimated from the seq.id. between the target and template sequences.

Most of these studies address quality issues regarding the model as a whole; however, because many applications of homology models depend on the quality of the biologically crucial parts of the protein [1,21,23,24], more recent work either includes specific analyses of these sub-structures[26,27,30,40] or is completely devoted to the same [34]. Among the points addressed is the hypothesis that some functional regions are better modelled than others because of their higher sequence and structure conservation [42]. Analyses of CASP experiments provide contradictory evidence either supporting [30] or rejecting [40] this hypothesis. Along another line, De-Weese and Moult [34] used CASP data to explore how ligand binding information can be obtained from comparative models. These authors analyzed the errors in protein-ligand contacts as well as the source of these errors (e.g. alignment problems, incorrect side-chain rotamers, etc). They found that when there are no alignment errors, comparative models provide a useful understanding of the interaction between the protein and its ligand, even at seq. id. levels of around 30%. Complementary to these CASP-based studies, recent large-scale studies of comparative models have also considered the quality of protein functional regions [26,27]. In these two studies, the authors describe the behaviour of several global, structure-dependent properties, such as accessible surface area and electrostatic potential, in comparative models [26,27]. In addition to examining these global properties, the authors also analysed the degree of conservation of protein clefts in terms of location and boundary residues. They reported that: (i) spurious clefts appear as seq.id. decreases; (ii) the more similar the target and template sequences, the more conserved the clefts; and (iii) clefts in models have a more rugged surface than in the experimental structure.

The work by De-Weese and Moult [34] and by Sanchez's group [26,27] provides a valuable, but still incomplete, picture of how the quality of functional cavities is preserved in comparative models. In the case of De-Weese and Moult's work [34], the reach of their results is limited by the following: the reduced number of proteins and models studied, 10 and 207, respectively; the consideration of only small molecule binding; and the fact that the analysis is based on the use of essentially one variable, distance root-mean-square deviation. Sanchez's group [26,27] studied a series of structure-based properties, including clefts. More precisely, in the case of clefts, their work was restricted mainly to the issue of the degree of their preservation between the experimental structure and the model. However, apart from the ruggedness study, no shape descriptors were used to specifically define cleft quality in protein models. In summary, and to the extent of our knowledge, there is no exhaustive study entirely devoted to assess how cleft structure varies in comparative models. Here we address this issue and examine the quality of clefts in protein models obtained at a range of target-template seq.id. levels, using six variables that cover various features of cleft structure. Although we provide data for the entire seq.id. range, we focused on the behaviour of comparative models in the medium (30% – 60%) and low (< 30%) ranges for the following reasons: (i) the quality of homology models above 60% seq.id. is usually high [1,23]; (ii) biochemical function above 60% seq.id. is usually conserved [43-45]; (iii) target selection protocols in structural genomics projects usually rely on a 30% seq.id. threshold to obtain a maximal coverage [6,46]; and (iv) comparative modelling is possible below 30% seq.id. because the protein structure is preserved below this threshold [43,47,48]. The study was carried out using 53507 comparative models (built with the standard modelling software MODELLER [49]) for 3802 protein CATH domains [50]. Our results provide a detailed and quantitative view of how cleft quality varies in comparative models and constitute a valuable guide for users of this structure prediction technique. More precisely, we (i) quantitatively show the dependence between several descriptors of cleft quality and seq.id. between target and template sequences; (ii) demonstrate that a certain number of good quality models up to 20% seq.id. can be found; and (iii) indicate that above 30% seq.id. cleft quality approaches that obtained when using the best possible alignments (structural alignments).

## Results and discussion

In Table 1 we show the range of seq.id. levels between target and template sequences for the models examined. While the whole sequence range was covered, the vast majority of the models clustered in the 0% – 60% interval, which constitutes the main focus of this study. Sequence alignments within this range showed a considerable number of non-aligned residues, which, in general, resulted in poorly modelled regions [23,24,51]. For this reason, we restricted our analysis to those clefts for which all contouring residues were aligned to a template residue.

**Table 1:** Sequence identity distribution for the target-template pairs.

**IDENTITY**	**ABSOLUTE FREQUENCY**	**RELATIVE FREQUENCY**
**0–10**	12650	23.64
**10–20**	28382	53.04
**20–30**	4868	9.10
**30–40**	1830	3.42
**40–50**	1069	2.00
**50–60**	650	1.21
**60–70**	172	0.32
**70–80**	16	0.03
**80–90**	12	0.02
**90–100**	25	0.05
**= 100**	3833	7.16

The domains chosen were distributed over the four CATH [50] classes (mainly-alpha: 24%; mainly-beta: 29%; alpha-beta: 45%; low secondary structure content: 2%), sampling 33 architectures and 390 topologies, thus giving a good coverage of the structure space of protein domains.

Clefts were computed for each experimental protein structure using SURFNET [52], which provides a list of clefts. We chose the largest cleft from this list because it is the one that is most likely to play a relevant functional/structural role. Furthermore, in whole proteins this cleft is usually associated with the biochemical function of the protein, by either participating in protein-protein interactions, or hosting the enzymes' active site [53,54]. In our case, in addition, because we considered protein domains, the largest cleft may also correspond to the locus of domain-domain interactions that determine the structure of the whole protein, thus playing an equally important structural role. However, given that smaller clefts may have a functional role in some cases, we also provide results for the top-five clefts.

### Shape changes

To explore how well clefts were reproduced in the models, we used six variables (see *Materials and Methods*): root-mean-square deviation (rmsd), normalized root-mean-square deviation (rmsd_100_), global distance test (GDT), protrusion index (cx), variation in accessible surface area (ΔASA) and contact number (ΔCN). Rmsd is widely applied in many areas of structural analysis, and in particular has been successfully used in the characterization of shape variations in binding sites [55,56], a problem formally analogous to that addressed in the present study. Rmsd_100 _[57] is a transformation of rmsd that eliminates the size dependence present in the latter and its use allowed us to exclude size biases from our results. GDT, developed within the context of CASP experiments [58], is a quality measure that helps to detect the presence of well preserved sub-structures in otherwise bad models, thereby helping to prevent the sensitivity of rmsd to outliers. Cx is a simple measure of the protrusion degree of protein atoms, related to the atomic environment, that can be used to characterise binding sites, cleavage sites, etc [59]. ASA [60] is a shape descriptor that has been extensively employed in protein structural analysis to describe, amongst others, energetic and functional features, such as atom-atom interactions [61,62], protein solvation [63,64], protein-protein interactions [65], etc. Finally, ΔCN, which is directly derived from ΔASA [66], provides an approximate idea of how comparative models preserve the capacity of cleft atoms to establish functional interactions.

#### Rmsd

Rmsd between the observed structure of the protein and the homology model was computed considering only the set of atoms defining the cleft in the former (see *Materials and Methods*). As a control, and to assess the limits introduced by the model building procedure itself, we employed the results of the auto-modeling process in which a model for the target protein was produced using its own experimental structure as template. Our results provide a basal line that corresponds to the limits of the modelling software – MODELLER [49] in our case- and includes the impact of the distinct approximations implicit in the different steps of the structure building process – e.g. the force field employed, the minimization protocol, the internal protein representation, etc.

**Figure 1:**
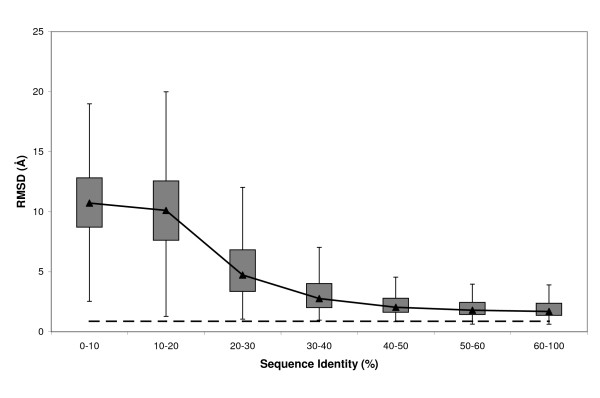
**Relationship between rmsd and seq.id**. This boxplot shows how cleft rmsd, computed (see *Materials and Methods*) after optimal superimposition between the experimental and modelled cleft structures of the target proteins, varies with target-template seq.id.. The dashed line represents the auto-model control (see *Materials and Methods*).

Cleft rmsd varied depending on the seq.id. between the target and the template sequences (Note that seq.id. was computed for whole sequences, it was not restrained to the cleft residues) (Figure 1). As expected, we observed that as the latter increased, cleft rmsd decreased, asymptotically approaching auto-modelling values. Most of the cleft models that showed poor conservation were found at seq.id. levels of less than 20%, where rmsd values were, in general, very high (more than 75% of the cases had rmsds over 7.6 Å). The number of good models increased with seq.id., and even in the 20% – 40% range well over 50% of the models showed clefts with rmsds below 5 Å. This observation indicates that even within this seq.id. range there are clefts that could be used for applications such as low-resolution compound screening, function identification, etc, as long as they can be singled-out from the background of low-quality cases. Over 40% seq.id., a plateau was reached, with ~50% of the cases clustering between 1.7 Å and 2.8 Å. These results indicate that even at very good seq.id. levels it may be difficult to reach the limits of the modelling method because of the effect of small sequence changes, the presence of bound ligands, crystal contacts, etc [24,26,27]. Thus even above 40% seq.id., standard modelling protocols may not be good enough for applications that require accurate models of the protein clefts of the target protein. A greater modelling effort – *e.g*. using molecular dynamics simulations [67], or conformational searches of the non-aligned regions, using *de novo *procedures like Rosetta [51], or modeling of the active site using specific templates [25], or, eventually, experimental determination of the target structure, may be required in these cases. Our results are partly consistent with the picture arising from the work of DeWeese and Moult [34], and show that in some cases good cleft models can be found even below the 30% threshold proposed for target selection protocols for structural genomics projects [6]. However, the sharp quality decrease observed for seq.id. levels lower than 20% indicates that below this threshold conventional sequence alignment methods in most cases will result in very poor models. Similar results were obtained when plotting rmsd as a function of cleft seq.id. instead of whole-protein seq.id. [see Additional file 1].

**Figure 2:**
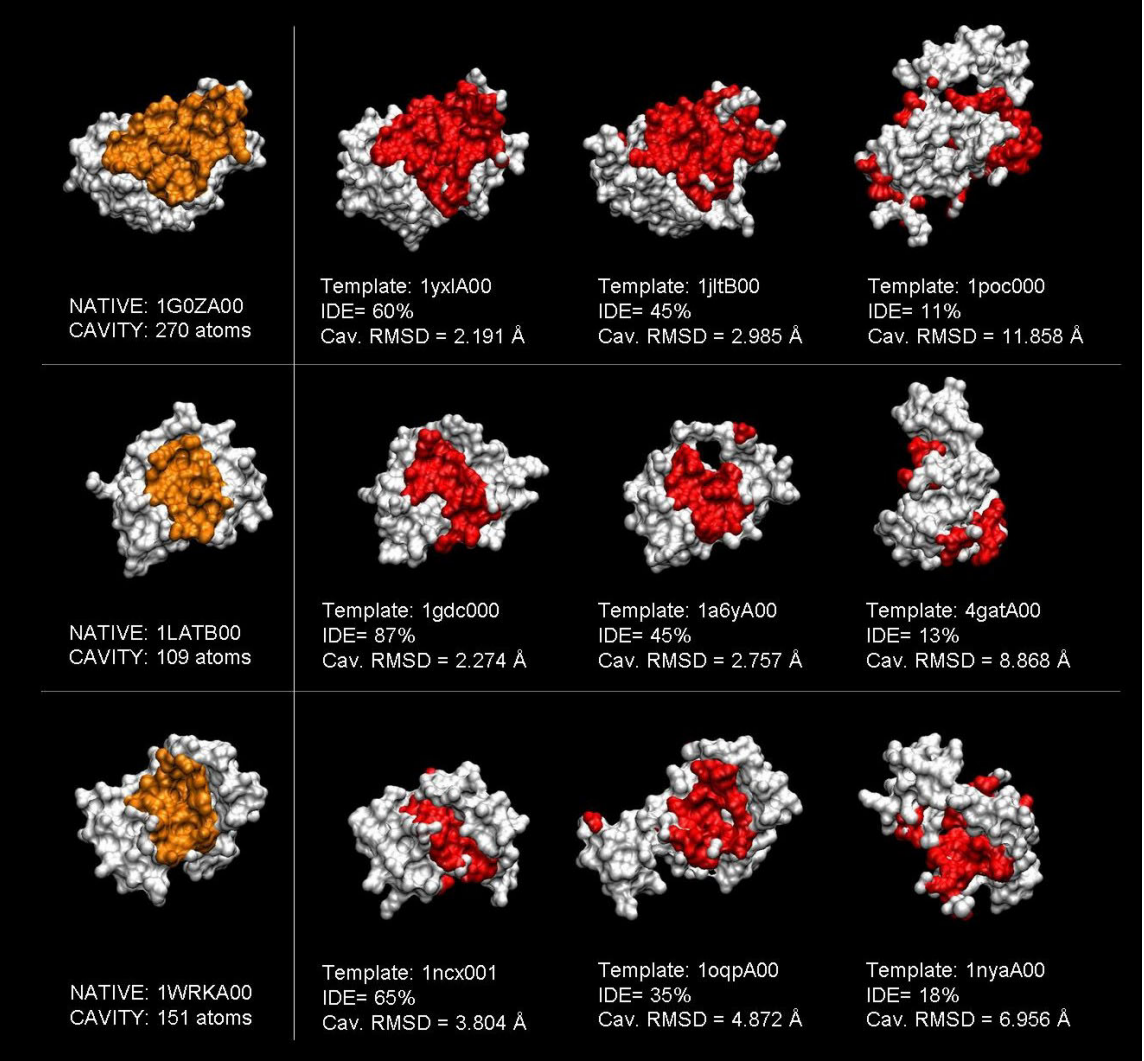
**Examples of the relationship between rmsd and seq.id**. For three cases (PDB codes: 1GOZ – *S. aureus *enterotoxin, 1LAT – *R. norvegicus *DNA binding domain of the glucocorticoid receptor, 1WRK – *H. sapiens *N-terminal domain of cardiac troponin C) we show how the quality of the largest cleft in the experimental structure decreases with seq.id. The atoms of the largest cleft in the experimental structures are shown in orange (structures in the left), while the same atoms are shown in red in the various homology models. Cleft quality becomes very poor at seq.id. below 30%, although even at higher seq.id. level some shape details are clearly lost. The distinct templates, as well as the target protein, are identified by their CATH [50] domain identifier, which comprises the four letters of the PDB code, a chain symbol, plus two digits indicating the domain.

**Figure 3:**
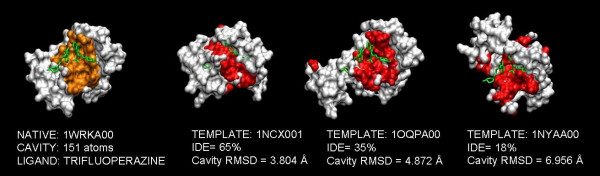
**Ligand binding in models at a range of seq.id. levels **Binding of trifluoroperazine to the N-terminal domain of cardiac troponin C (PDB code: 1WRK). The experimental structure of the complex is shown on the left, with the two trifluoroperazine molecules shown in green and the cleft atoms highlighted in orange. The latter are shown in red in different models of the protein, while the trifluoroperazine molecules (green colour) are kept in the same orientation as in the experimental structure. We can see that in this case, even for good seq.id., protein-ligand contacts are poorly reproduced.

To illustrate the rmsd results with specific examples, Figure 2 shows three cases where the first cleft observed in the target's experimental structure is highlighted in models obtained at distinct seq.id. levels. While the global shape and location of the cleft were preserved above 30% seq.id., this was not the case for seq.id. below this threshold. The impact that shape changes may have on the modelling of protein-ligand interactions is exemplified in Figure 3, where the ligand (trifluoroperazine) is shown with the same orientation it has in the experimental structure of the complex. Even at high seq.id., the structure of the cleft may not be of sufficient quality to properly reproduce the protein-ligand interaction pattern.

**Figure 4:**
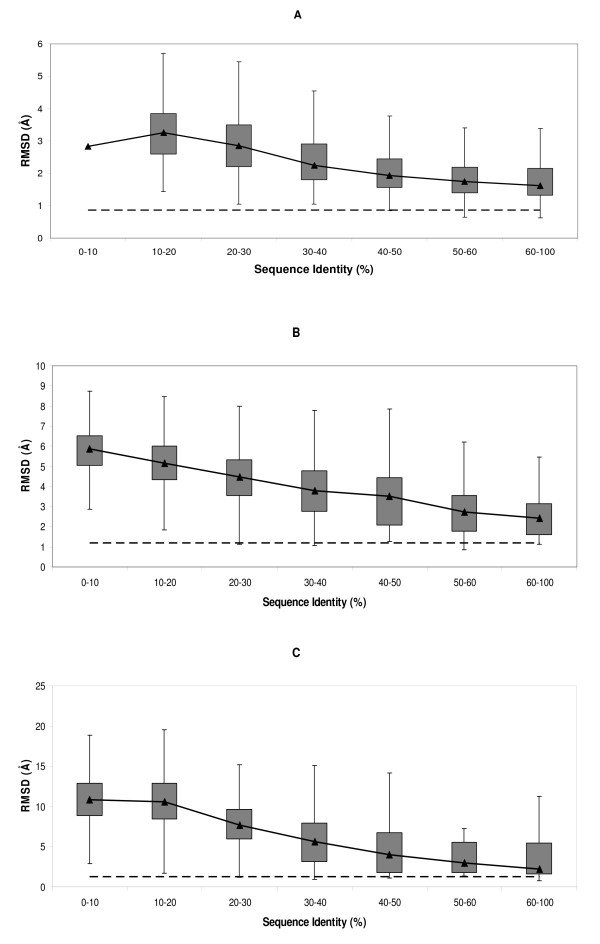
**Cleft vs. backbone rmsd**. The three boxplots correspond to the distributions of cleft rmsd relative to target-template seq.id. for models with (A) high (0 Å – 3 Å), (B) medium (3 Å – 6 Å) and (C) low (≥ 6 Å) backbone accuracy (computed as the rmsd of the C_α _trace between experimental and modelled structures of the target). Please note the scale change between figures. In all three cases the dashed line represents the auto-model control (see *Materials and Methods*).

To complete the previous view, we explored the relationship between cleft and backbone quality. This is an important point, particularly when considering further refinement of the models with techniques such as molecular dynamics, which, *a priori*, treat all protein atoms equally. When sufficiently large and in absence of specific restraints, the poorly modelled parts may prevail over the better parts, thus resulting in an effective degradation of the latter. This may occur when attempting to refine comparative models in which functional clefts are better modelled than the rest of the protein because of functional constraints [30,68]. In our analysis we divided the previous cleft rmsd data in three classes, on the basis of backbone quality (measured using C_α _rmsd): high (0 Å – 3 Å), medium (3 Å – 6 Å) and low (≥ 6 Å). We found (Figure 4) that above 30% – 40% seq.id. a considerable proportion of the clefts showed an rmsd lower than the corresponding backbone rmsd, particularly for high and medium quality backbones. Two main opposing factors are likely to contribute to this trend: the existence of functional constraints acting on the first cleft and the presence of poorly modelled parts in the rest of the structure. The former would result in better cleft rmsd and the latter in poorer backbone rmsd. Regardless of the case, our results suggest that subsequent refinement of initial models obtained within the 40% – 100% seq.id. range may require the application of several restraints to the cleft contouring atoms, at least in the first steps, in order to preserve the initial cleft quality. For lower seq.id. levels, overall model refinement could eventually result in an improvement in cleft quality.

#### Rmsd_100_

**Figure 5:**
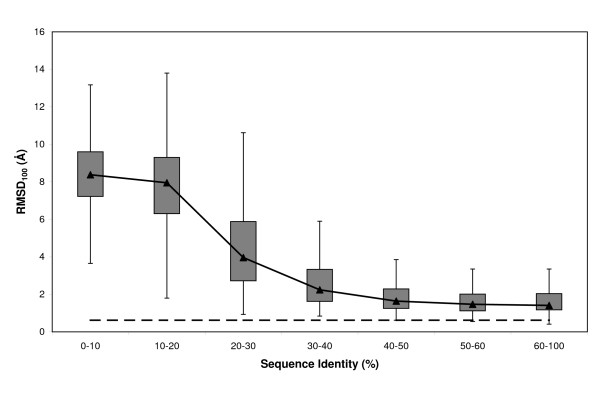
**Relationship between rmsd_100 _and seq.id**. Boxplot of the rmsd_100 _distribution relative to seq.id. We used rmsd_100 _[57] instead of rmsd to eliminate the effect of cleft size on our analyses. The dashed line represents the auto-model control (see *Materials and Methods*).

The meaning of rmsd as a quality measure depends on the size of the elements compared [57,69-71], that is to say, while 4 Å rmsd may indicate high similarity when comparing 1000 residue proteins, it may suggest poor resemblance if small active sites are compared. Because the clefts considered in this study were of distinct sizes, we used rmsd_100 _[57], a normalized rmsd which is independent of size. The behaviour observed for rmsd_100 _(Figure 5) was comparable to that found for raw rmsd (Figure 1), showing the same asymptotical trend towards auto-modelling values and the large variability within seq.id. bins. In addition, we also found the quality transition between 10% and 30% seq.id. present in the rmsd data (Figure 1). This confirms the independence of our main results from cleft size.

#### GDT

**Figure 6:**
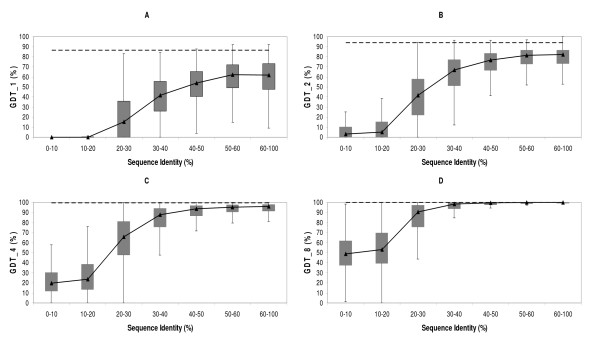
**GDT analysis**. The four boxplots show the distributions of (A) GDT_1, (B) GDT_2, (C) GDT_4 and (D) GDT_8, respectively, relative to target-template seq.id.. GDT values are related to the presence of sub-structures modelled below a certain distance threshold (see *Materials and Methods*). In all four cases the dashed line represents the auto-model control (see *Materials and Methods*).

GDT is a summary measure directly related to the presence of quality/well-preserved sub-structures within the model, and works by identifying the percent of atoms modelled below a given distance threshold [58]. Application of a range of distance thresholds provides a complete view of how quality varies within the predicted structure; in our case we used four commonly used thresholds [58], 1 Å, 2 Å, 4 Å and 8 Å, which result in four GDT values, GDT_1, GDT_2, GDT_4 and GDT_8. Smaller thresholds were discarded as we focused mainly on seq.id. below 60%, where models tend to be of poor quality. After considering the results for the four thresholds (Figure 6) together, cleft models were divided into two classes on the basis of seq.id.. Above 30% seq.id, a considerable proportion of the clefts showed large GDT_1 and GDT_2 values, indicating the presence of high-quality sub-structures. Because of the *a priori *value of these parts, this result supports the use of post-modelling analysis for their identification (e.g. using specific energy functions, residue conservation or literature analysis), as they may provide a good starting point for further refinement of the cleft model. In contrast, models below 30% seq.id. showed few or no high quality sub-structures (Figures 6A and 6B). In the medium quality threshold (Figure 6C), corresponding to GDT_4 values, a non-negligible fraction of cleft models below 30% seq.id. showed sub-structures of such quality. These sub-structures may not be useful for highly demanding applications, such as drug design, but may be a reasonable starting point for further refinement of the model, or provide a coarse-grained view of some aspects of protein function, e.g. rough shape of the binding site [1].

**Figure 7:**
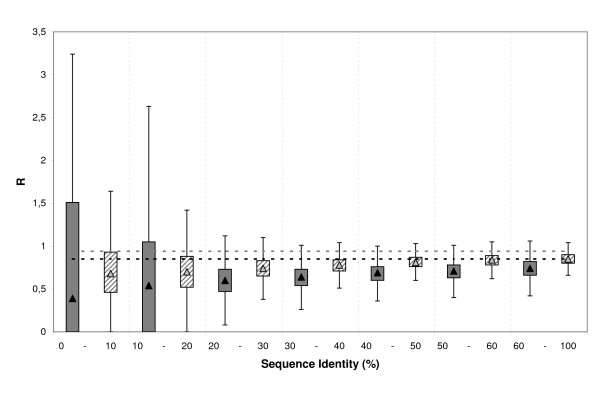
**Side-chain contribution to cleft quality**. The boxplot shows the distribution of R values relative to target-template seq.id.. R is the ratio between the percentage of side-chain atoms in the list of atoms contributing to a given GDT and the percentage of side-chain atoms in the cleft's set of contouring atoms.. The figure shows the distributions corresponding to GDT_1 (grey boxes) and GDT_2 (dashed boxes) values. The dashed lines correspond to the respective auto-model controls: dark grey for GDT_1 and light grey for GDT_2. Vertical dashed lines are used to separate the seq.id. bins.

Side-chain atoms constitute a large fraction of the set of cleft contouring atoms. Because side-chains are usually hard to model [21], we studied their contribution to cleft quality. To this end, for each cleft we computed the ratio, which we called R, between two percentages: the percentage of side-chain atoms in the list of atoms contributing to a given GDT (GDT_1, GDT_2, etc) and the percentage of side-chain atoms in the cleft's set of contouring atoms. If, side-chain and main-chain atoms are modelled with equal accuracy R will be equal to one. However, if side-chains are poorly modelled than main-chain atoms R will be lower than one (the opposite is true when side-chain atoms are better modelled than main-chain atoms). We focused our analysis on GDT_1 and GDT_2 values because these identify high-quality modelled sub-structures. The results for GDT_3 and GDT_4 are provided as additional file [see Additional file 2]. When we plotted the distribution of R values (Figure 7), we observed that auto-modelling R values were slightly lower than 1, indicating that even in this ideal situation the modelling of side-chain atoms is poorer than main-chain atoms. If we now consider our set of models, in general, R values were below 1, but approached asymptotically auto-modelling values as target-template seq.id. increased. This observation indicates that main-chain atoms make a stronger contribution to the best-modelled parts of clefts; however, as seq.id. increased side-chain building improved and their contribution almost reached the limits imposed by the modelling software. The large fluctuations in R observed in the 0% – 30% seq.id. range (Figure 7), in particular for GDT_1, were probably a consequence of inaccurate main-chain modelling, which in turn resulted in an almost random building of side-chains. As alignment quality improved so did backbone accuracy, thereby leading to better built side-chains, which in turn resulted in better R values for seq.id. above 30%, an improvement that was particularly notable for GDT_1. On the basis of these results, an increase in cleft quality could be expected after improving the side-chain modelling, for example using the SCRWL package [72]. However, results from Sanchez's group [27] indicate that surface properties are not particularly sensitive to better side-chain modelling, and cannot be improved by the single use of SCRWL [72]. Instead, these authors report that improvements in the force field, e.g. better solvation term, may be required to correctly model surface properties [27]. An alternative option to extract more information from available cleft models, or at least to explore the cleft's conformational space, would be the use of restrained molecular dynamics [25]. In this approach all model atoms are frozen except those defining the protein's active site, which are allowed to move freely, subject to covalent restraints with the rest of the structure. The resulting trajectory gives an approximate view of correlations between residues, cleft volume, etc, which may be useful in the design of coarse-grained probes to screen small molecule 3D databases [25].

#### Cx

cx is a volume ratio (see *Materials and Methods*) that gives a local measure of the atomic environment that can be related to function [59]. We computed the percentage of cleft atoms for which the cx value varied between the observed and the model structures and examined how this number varied with target-template seq.id.. To exclude noise corresponding to small experimental fluctuations, we followed a simple protocol (see *Materials and Methods*). We first obtained a set of 223 structure pairs with each pair member corresponding to a different experimental version of the same structure. We then computed the difference in cx for all pairs of equivalent atoms and plotted the resulting distribution (data not shown). For over 99% of the cases, the difference in cx was between -1 and 1. On this basis we considered that: for any given atom cx had varied between the experimental and the model structures when the difference in cx was larger than 1 in absolute value.

**Figure 8:**
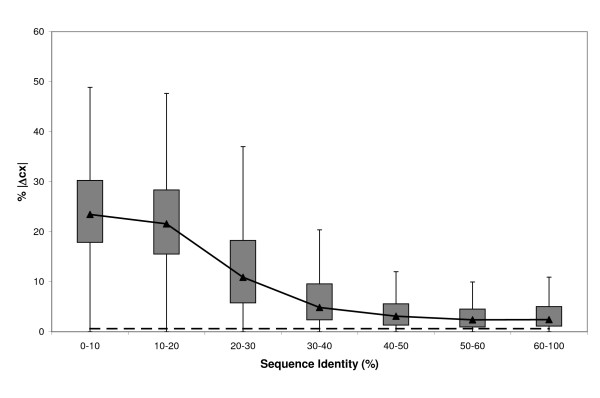
**cx [59] conservation in comparative models**. Boxplot showing the distribution, relative to seq.id., of the percentage of cleft atoms with different cx values (|Δcx| > 1, see *Materials and Methods*) between the experimental structure and the model. cx [59], or protrusion index, is an atomic-level shape descriptor that can be used to identify functional regions in proteins. The dashed line represents the auto-model control (see *Materials and Methods*).

The atomic local structure of cleft models obtained at seq.id. above 30% – 40% was almost equally well preserved along the whole seq.id. range (Figure 8). In contrast, for seq.id. below 20% – 30% the percentage of atoms with cx values varying between observed and model structures increased substantially, showing a transition similar to that found for rmsd data (Figure 1). This finding indicates that cleft structures for models obtained at low seq.id levels show large changes in both their global (rmsd data, Figure 1) and local (cx data, Figure 8) features. The cx result was also consistent with the lack of common sub-structures observed in GDT_1 and GDT_2 (Figure 6) analyses. Taken together, these results indicate that model refinement at this seq.id. requires large conformational searches, or introduction of external restraints (taken either from the literature, or from additional experiments) in order to obtain true improvements.

#### ΔASA and ΔCN

**Figure 9:**
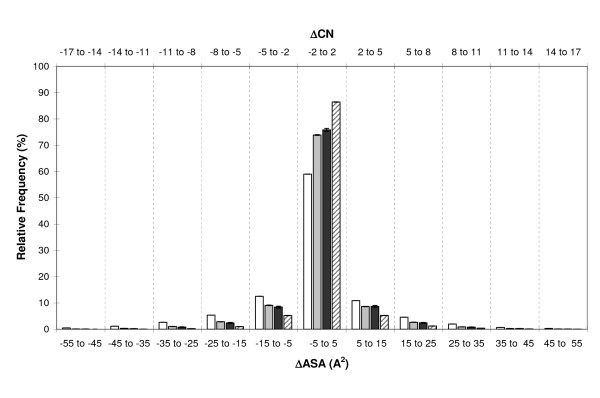
**ASA conservation in comparative models**. The figure shows the distribution of atomic ΔASA (ΔASA = ASA_experimental _– ASA_model_) for cleft atoms and four cases: low- (< 30% seq.id, white), medium- (30% ≤ seq.id. < 60%, light grey) and high-quality (> 60% seq.id., dark grey) models, and auto-models (dashed). On the top x-axis we display the number of atom-atom contacts equivalent to the ΔASA value in the bottom x-axis, estimated using [66]: ΔCN ~ 0.31ΔASA. Vertical dashed lines are used to separate the ΔASA bins.

To complete the picture, we explored the changes in atomic ASA experienced by clefts in comparative models. This analysis complements previous analyses as changes in ASA are related to protein energetics, e.g. solvation free energy [63] or free energy of atom-atom interactions [61,62]. This analysis provides an approximate idea of how well we can model native interactions of the target protein with other molecules [65] – either quaternary structure partners, small substrates or designed drugs. To this end, we divided our set of models in three quality groups: low (< 30% seq.id.), medium (30% – 60% seq.id.) and high (≥ 60% seq.id.). For each of these quality bins, we computed the change in ASA for all atoms of the largest cleft (Figure 9). In accordance with our previous results, ASA changes (i) tended towards the auto-modelling values as seq.id. increased; (ii) were larger the lower the quality of the model; and (iii) the distributions for medium and high quality models differed substantially from that of low quality models. The latter was more spread over the ΔASA range, a result that completes cx results (Figure 8), thereby confirming the presence of significant local changes in the atomic environment. We also observed that changes in ASA values were evenly distributed around zero, indicating that the modelling protocol introduces no substantial biases towards exposing or burying cleft atoms.

As mentioned previously, a number of applications of comparative modelling, like drug design or study of enzyme-substrate interactions, require accurate modelling of native atomic interactions between the target protein and another molecule (either a small substrate or a macromolecule). To provide an estimate of how modelling of these interactions may vary in comparative models, we used an additional parameter, ΔCN (changes in contact number), which is computed from ΔASA using an approximated relationship proposed by Colonna-Cesari and Sander [66]: ΔCN ~ 0.31ΔASA. ΔCN gives a rough idea of how changes in solvent accessibility can modify the ability of cleft atoms to establish interactions with other molecules.

We found (Figure 9) that even for high-quality models almost 25% of cleft atoms had ΔCN values around three. This indicates that these atoms had either gained (ΔCN E 3) the ability to establish three non-native interactions or lost (ΔCN E-3) their ability to establish three native interactions, on average. Furthermore, while this situation was comparable for medium-quality models, for low-quality models the figure rose to over 40% of the cases.

### Factors affecting cleft quality

Finally, we studied the effect of several factors contributing to cleft quality, focusing on two related issues: (i) the effect of non-aligned cleft contouring residues; and (ii) the maximal improvement we could obtain when optimal target-template alignments were available. We also examined whether cleft quality was affected by differences in protein fold, or cleft rank (using the five largest clefts of a protein, instead of the largest one), although these results are provided separately as additional files [see Additional files 3 and 4]. To take into account the size effect, we used rmsd_100 _in all cases.

**Figure 10:**
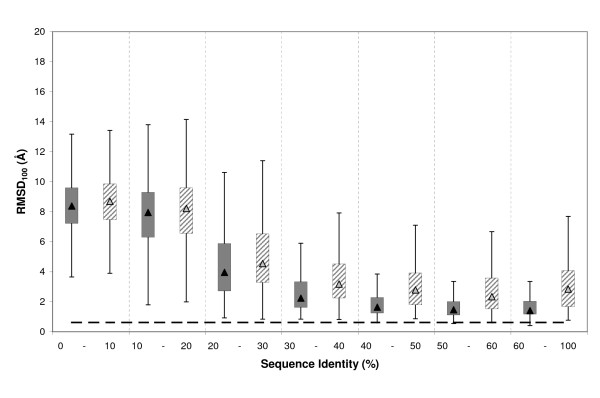
**Effect of non-aligned residues**. Boxplot for the distribution of rmsd_100 _values for clefts with 100% (grey boxes) or more than 75% (dashed boxes) of their residues aligned, respectively. The dashed line represents the auto-model control (see *Materials and Methods*). Vertical dashed lines are used to separate the seq.id. bins.

Non-aligned residues lead to poorly modelled regions [23,24,51]. We therefore focused on clefts in which all residues were aligned. However, in some cases when the number of non-aligned residues is relatively small, the restraints imposed by the rest of the structure [73] may result in acceptable models for this structural region. To explore this idea, our analysis included all models with a small fraction (≤ 25%) of non-aligned residues affected. Figure 10 shows a comparison of cleft models with 100% or at least 75% of residues aligned to the template, respectively. The latter tended to show poorer rmsd_100 _values in the medium (30% – 60%) and high seq.id. (> 60%) range. However, the differences were not so large as to exclude the usefulness of these models. In the low identity range (0% – 30%), alignment quality was too low to result in reasonable cleft models, even when all residues were aligned.

**Figure 11:**
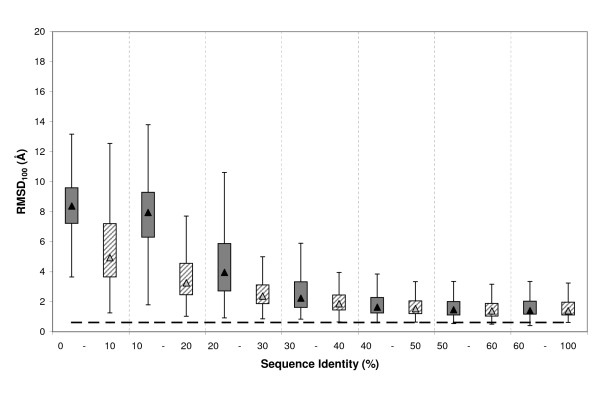
**Sequence vs. structure alignments**. Boxplot for the distribution of rmsd_100 _values relative to target-template seq.id. for models obtained from sequence (grey boxes) and structure (dashed boxes) alignments, respectively. The dashed line represents the auto-model control (see *Materials and Methods*). Vertical dashed lines are used to separate the seq.id. bins.

Within this context we attempted to establish the maximal quality that can be reached by improving sequence alignment. This point is of particular relevance since it may help the user of comparative modelling to determine whether it is worth investing time and effort in ameliorating the target-template alignment. To this end, instead of sequence alignments we used structure-based alignments as input to MODELLER. These alignments were obtained, for all target-template pairs, using the MAMMOTH suite [74,75] and correspond *a priori *to the best alignment obtained between two sequences. When comparing the rmsd_100 _distributions for models obtained using either sequence or structure alignments (Figure 11) we distinguished two scenarios. Below 30% seq.id., cleft models derived from structural alignments were clearly better that those obtained from sequence alignments. This finding shows that, in this case, improving sequence alignments is beneficial. However, above 30% seq.id., sequence-sequence alignments improved and cleft quality started to depend more on having all cleft residues aligned (Figure 10), or on factors related to the template structure, such as crystal contacts, presence of bound ligands, etc, mentioned in previous sections [24,26,27].

## Conclusion

Here we provide a quantitative view of how the quality of protein clefts varies in comparative models, depending on the seq.id. between the target and template sequences. Our results show how cleft quality – measured using rmsd, rmsd_100_, GDT, cx, ASA and contact number- is related to target-template seq.id.. When considered together, these analyses consistently show that below 20% seq.id. cleft quality undergoes a clear decrease, both from a global (Figure 1) as well as from a local point of view (Figures 6, 8 and 9). This finding suggests that even between 20% and 30% seq.id., useful models of protein clefts can be obtained, although the use of quality assessment tools is strongly advised, due to the important proportion of poor models within this seq.id. range. Once identified, the cleft model may be subject to subsequent refinement steps aimed at improving quality, e.g. using global model refinement (taking advantage of the better backbone quality, Figure 7), although the greatest improvement is likely to result from the use of good alignments (Figure 11). Above 30% – 40% seq.id., the main restriction to model quality is determined by the template selected (Figure 1). Within this seq.id. range, overall backbone structure tends to deteriorate more than cleft structure, probably because of functional restraints on the latter. Therefore, further model refinement should probably freeze, at least partly, the structure of the cleft, to prevent degradation. Overall, our work goes beyond previous studies [26,27,34] presenting a complete view of how the structure of protein clefts varies in comparative models, which constitutes a useful guide for researchers interested in the study of protein function using comparative modelling methods.

## Methods

Here we explored the level of preservation of protein clefts in comparative models. While the latter can be obtained using templates with varying degrees of sequence similarity from the target, our interest focused mainly on complete coverage of the seq.id. range below 60%, for the reasons mentioned above. This decision determined our selection strategy of targets and templates, which was designed to provide a large number of models within this seq.id. range. Nonetheless, models were also obtained at higher seq.id. to confirm the consistency of the trends observed.

### The target-template pairs

Homology, or comparative, modelling methods have the capacity to produce a 3D structure for any target protein provided that structural information is already available for at least one of its family members [1,23,24], usually called the template(s). Thus the starting point of our study was to build a list of target-template protein pairs that covered the desired seq.id. range. The difference, in our case, was that the structure of the target protein had to be known in order to allow the assessment of protein clefts variation among comparative models of distinct quality. To this end, we used the CATH database [50], version 3.0.0.. This database is a domain database in which whole protein structures have been previously separated into their constituting domains. While the use of protein domains did not affect the results of our analysis, it slightly affected the functional/structural meaning of the cavities considered. As explained above, the largest cleft was selected for our analysis because it usually coincides with the protein functional locus [53,54]. However, because we are dealing with domains, some of these clefts may appear only after separating interacting domains from the same protein. Their value, for the purpose of our research, is similar to that of other functional clefts, as they play a vital role when docking independently modelled domains to build the structure of multi-domain proteins [76].

CATH provides a hierarchical classification of protein domains [50] with a range of levels that go from very broad – like the Class level- to very fine, sequence family levels. Among the latter is the O-level, in which domains with seq.id. higher than 60% are grouped, and which was used as a starting point in our study. Indeed, our set of target-template pairs corresponded to all possible pairs of O-level representatives belonging to a given H-level (Homologous Superfamily level), for all H-levels. The list of O-level representatives was obtained from the CATH server [50], and was subsequently filtered: we excluded all discontinuous CATH domains, structures not considered as true folds in SCOP [77] (version 1.67), and all protein domains with missing atoms, or main-chain discontinuities. The resulting number of structures was 3802 and the list of target-template pairs had 90948 pairs. The latter were used as input to the program MODELLER and resulted in 88410 models, as there was a small fraction of target-template pairs for which no alignment could be produced. A final filter was implemented to leave only those cases for which all residues from the target's largest cleft (see below) were aligned to template residues. The final number of target-template pairs was 53507 (A list with the pdb codes for all these pairs is provided target-template pairs is provided as additional file [see Additional file 5].

### The homology modelling protocol

The homology models were obtained with the standard program MODELLER [49], using the sequence alignment between the target and template sequences as input. The latter were extracted directly from the CATH [50] file CathDomainDescriptionFile.v3.0.0, and aligned using the ALIGN option from MODELLER [49] which implements a global dynamic programming algorithm with afine gap penalties [78]. The models were built using MODELLER's default parameters.

To assess the quality limits imposed by the comparative modelling software, we used a set of models obtained using the experimental structure of the target protein as template, which amounted to a total of 3797 models (five less than the 3802 due to small discrepancies between the PDB sequence and that given in the file CathDomainDescriptionFile.v3.0.0). This auto-model control gives a good idea of how the bias introduced in the distinct structural features (e.g. side-chain torsional angles, atom-atom contacts, etc) by the explicit and implicit approximations in the modelling package affect the final quality of the model.

Alignment accuracy is one of the most important issues in homology modelling [1,21,23,24], as it has a strong effect on the final quality of the model (*e.g*. alignment errors are essentially unrecoverable). In our study, where the goal was to assess the impact of conventional modelling on cleft quality, we generated sequence alignments with the ALIGN option from the MODELLER package [49], as done by Sanchez's group [27] in their work on the impact of comparative models on structure-derived properties. It must be noted, however, that the performance of dynamic programming algorithms, such as that implemented in ALIGN, decreases for seq.id. below 20% – 30% [79]. For this reason, the results shown in our study for seq.id. below 30% constitute a lower bound estimate of cleft quality. There are currently several alternatives to conventional dynamic programming [80] to obtain sequence alignments within this seq.id. range. However, it is unclear which is the best [80], and proper assessment of these alternatives is a difficult task to which much research effort is devoted and beyond the scope of the present study. Instead, following Sanchez's approach [27], we used structure-based alignments to show how an increase in alignment accuracy may improve cleft models. In our case the structure alignments were obtained using the MAMMOTH software suite [74,75]. The final number of models derived from these alignments, 89563, was slightly higher because MAMMOTH aligned some of the target-template pairs that were too difficult to align using sequence information alone. Again, for our analyses we only considered those models for which all cleft residues from the target were aligned to template residues.

### Sequence identity

As a reference for the user of homology modelling methods, we related all our results to the similarity between the target and template sequences, as provided by the MODELLER package, which is equal to: (number of identical residues)/(number of residues of the shortest sequence).

### Cleft computations

Clefts were obtained using the standard software SURFNET [52] which, for a given a protein, gives a list of clefts, each defined by a set of contouring atoms. We used the number of contouring atoms of a cleft as a measure of its size. For all our analyses, we focused on the largest cleft, except in one case [see Additional file 4] where we examined the five largest clefts.

### Changes in protein clefts

We used 6 parameters to characterize changes in protein clefts: root-mean-square deviation (rmsd), normalized root-mean-square deviation (rmsd_100_), global distance test (GDT), protrusion index (cx), variation in accessible surface area (ΔASA) and contact number (ΔCN). Unless otherwise stated, these parameters were computed using only the subset of protein atoms defining the chosen cleft in the target's experimental structure. For example, if for a given protein this cleft was defined by atoms a_12_, a_23_, a_34_, ..., a_332_, the rmsd computation between the experimental and the modelled structure was restricted to these.

#### Rmsd

We used the coordinates rmsd [81] as a quality measure of the clefts resulting from the modelling process. This rmsd is usually computed using all protein atoms, or main-chain atoms, etc. However, in our case we used the list of contouring atoms of the target's largest cleft. In some cases (Figure 4) we also obtained the rmsd using all the protein C_α _atoms, to relate cleft and backbone qualities.

#### Rmsd_100_

The normalized rmsd, rmsd_100_, is obtained from conventional rmsd using the following formula [57]: rmsd/[1 + 0.5ln(N/100)], where rmsd is the non-normalized value (obtained as explained in the previous section), and N is the number of aligned residue pairs. rmsd_100 _is independent of size [57] and therefore allows comparison of changes observed for clefts of different sizes on the same scale.

#### GDT

The global distance test is a measure used when comparing two structures. It allows the identification of common sub-structures between them. This measure corresponds to the percentage of aligned atoms that are at a distance lower than a given threshold. Here we used four thresholds, 1 Å, 2 Å, 4 Å and 8 Å, which are typically applied to assess structure predictions. GDT was computed for each threshold following an iterative procedure [58]:

a- compute the optimal superimposition [81] between the cleft atoms in the experimental and model structures, as explained in the **rmsd **section

b- find all aligned atom pairs at a distance lower than the threshold

c- obtain the optimal superimposition using only the atom pairs obtained in step b.

d- repeat steps b and c until no changes are observed in the pairs list during two iteration cycles.

#### Cx

cx [59] is a parameter that provides a fine-grained, local view of atomic environment. It is equal to the ratio between free and occupied volume within a sphere (10 Å radius) centered in each heavy atom. cx varies between 0 and 15, with large values corresponding to protruding atoms that may either be involved in protein-protein interactions, or correspond to proteolysis sites. cx was computed with the program developed by Pintar and colleagues [59].

For a given atom, when comparing cx values between structures, we may find small fluctuations that are probably meaningless. To establish a threshold beyond which variation in cx values may be relevant, we compared a set of pairs of replicas of the same structure, obtained under different experimental conditions. This pairs list was obtained by clustering all PDB [82] structures from the version of May 25th, 2007. We implemented a series of filters to exclude: theoretical models, modified residues, incomplete residues, missing and/or unknown residues, extreme experimental conditions (e.g. high or low pressure, etc), and mutants. After applying these filters, we then clustered the accepted proteins with Cd-hit [83] and eliminated those cases for which there were length differences between cluster members. The final list comprised 223 pairs of equivalent protein structures. For each protein atom we then computed, Δcx, the difference in cx between replicas. We found (results not shown) that over 99% of Δcx values clustered between -1 and 1. On this basis we imposed that only atoms with variations in cx larger than 1 in absolute value between the experimental and model structure would be taken into account.

#### ΔASA

The atom ASA was computed using the program NACCESS [84] with probe radius equal to 1.4 Å. It is a shape descriptor related to the capacity of atoms and residues to interact with their environment.

#### ΔCN

Change in contact number is derived from ΔASA following the study of Colonna-Cesari and Sander [66]: ΔCN ~ 0.31ΔASA. **ΔCN **provides a coarse-grained view of how the interaction capacity of a given atom varies as a result of changes in the model.

### Graphical representation

To plot the large number of data resulting from our analyses we mostly used boxplots instead of dotplots to avoid the overplotting problem that affects the latter [85]. Boxplots are usually employed to represent continuous variables and facilitate comparison between distributions [85]. Apart from the median, which is represented as an independent point with a special symbol (a triangle in our case), there are three main features in the boxplots: the central box, the "whiskers" and the outliers. The central box goes from the first (25th percentile) to the third quartile (75th percentile). One "whisker" starts at the first quartile and goes down the graph; the other "whisker" starts at the third quartile and goes to the top of the graph. The length of these "whiskers" is equal to the minimum between the respective extreme values and 1.5 times the interquartile range (the difference between the 75th and the 25th percentile values). Outliers are plotted as separate points. For clarity, we omitted outliers, but no conclusion was affected by their absence.

## List of Abbreviations used

ASA: accessible surface area

CN: contact number

cx: protrusion index

GDT: global distance test

GDT_1, GDT_2, GDT_3 and GDT_4: global distance tests computed for 1 Å, 2 Å, 4 Å and 8 Å distance thresholds, respectively

rmsd: root-mean-square deviation

rmsd_100_: normalized root-mean-square deviation

seq.id.: sequence identity

R: ratio between number of cleft side-chain atoms contributing to a given GDT (e.g. GDT_1, GDT_2, etc) and total number of cleft side-chain atoms.

## Authors' contributions

DP built the database of models and did most of the analysis. SL contributed to the cleft computations and analyses. XdC conceived the study, designed most of the testing and wrote the article. All the authors read and approved the final manuscript.

## Supplementary Material

Additional file 1 RMSD vs. CLEFT SEQUENCE IDENTITY. The boxplot shows how cleft rmsd varies with target-template seq.id., with the latter computed for cleft residues only. The results are very similar to those shown in Figure 1 of the main body of the article, with rmsd improving as seq.id. approaches 100%; the quality transition observed below 20% is only slightly smoother. This result is in accordance with the study by DeWeese-Scott and Moult[34]. Click here for file

Additional file 2 SIDE-CHAIN CONTRIBUTION TO GDT_4 AND GDT_8 vs. SEQUENCE IDENTITY. To assess the contribution of side-chains to cleft models we computed R, the ratio between the percentage of side-chain atoms in the list of atoms contributing to a given GDT (GDT_1, GDT_2, etc) and the percentage of side-chain atoms in the cleft's set of contouring atoms. In the main body of the article we discuss the results for GDT_1 and GDT_2. In this figure we show the boxplot for GDT_4 and GDT_8. For GDT_4, which is associated with medium quality sub-structures, we observe that most of the models have R values below 1, thereby reflecting that main-chain atoms are modelled with better accuracy than side-chain atoms. For GDT_8, we observe that most R values are between 0.8 and 1, indicating that at this low quality level, almost all cavity atoms are included in the cleft model. Vertical dashed lines are used to separate the seq.id. bins. Click here for file

Additional file 3 Dependence of results on protein family. Several CATH[50] families are large and naturally contribute a larger number of models than smaller families to our results. To examine whether the latter show a specific behaviour, we reproduced the analysis of Figure 5 for families contributing less than 100 models each (dashed boxes). We subsequently compared the resulting rmsd_100 _distribution with that of the whole set of models (grey boxes). No substantial differences are observed between sets. Vertical dashed lines are used to separate the seq.id. bins. Click here for file

Additional file 4 Dependence of results on cavity rank. In some cases it may occur that the protein function locus is located in a secondary cleft rather than in the largest cavity. To explore the quality with which smaller clefts are modelled, we show the comparison between the rmsd_100 _distributions for the largest cleft (grey boxes) and the top five clefts (dashed boxes). The latter, particularly for seq.id. below 30%, tend to have poorer qualities thus suggesting that secondary clefts are reproduced with lower quality in comparative models. This poorer reproduction is probably because these cavities have a smaller number of matching residues in the target-template alignment. Vertical dashed lines are used to separate the seq.id. bins. Click here for file

Additional file 5 List of target-template pairs used. The columns in the file correspond to the: first four CATH[50] numbers of the template and to the CATH[50] codes of the target and template, respectively. Click here for file
