# Supplementary figures and images for: Preservation of protein clefts in comparative models
Source: BMC Struct Biol. 2008 Jan 16;8:2. doi: 10.1186/1472-6807-8-2 (PMC2249585; doi:10.1186/1472-6807-8-2)

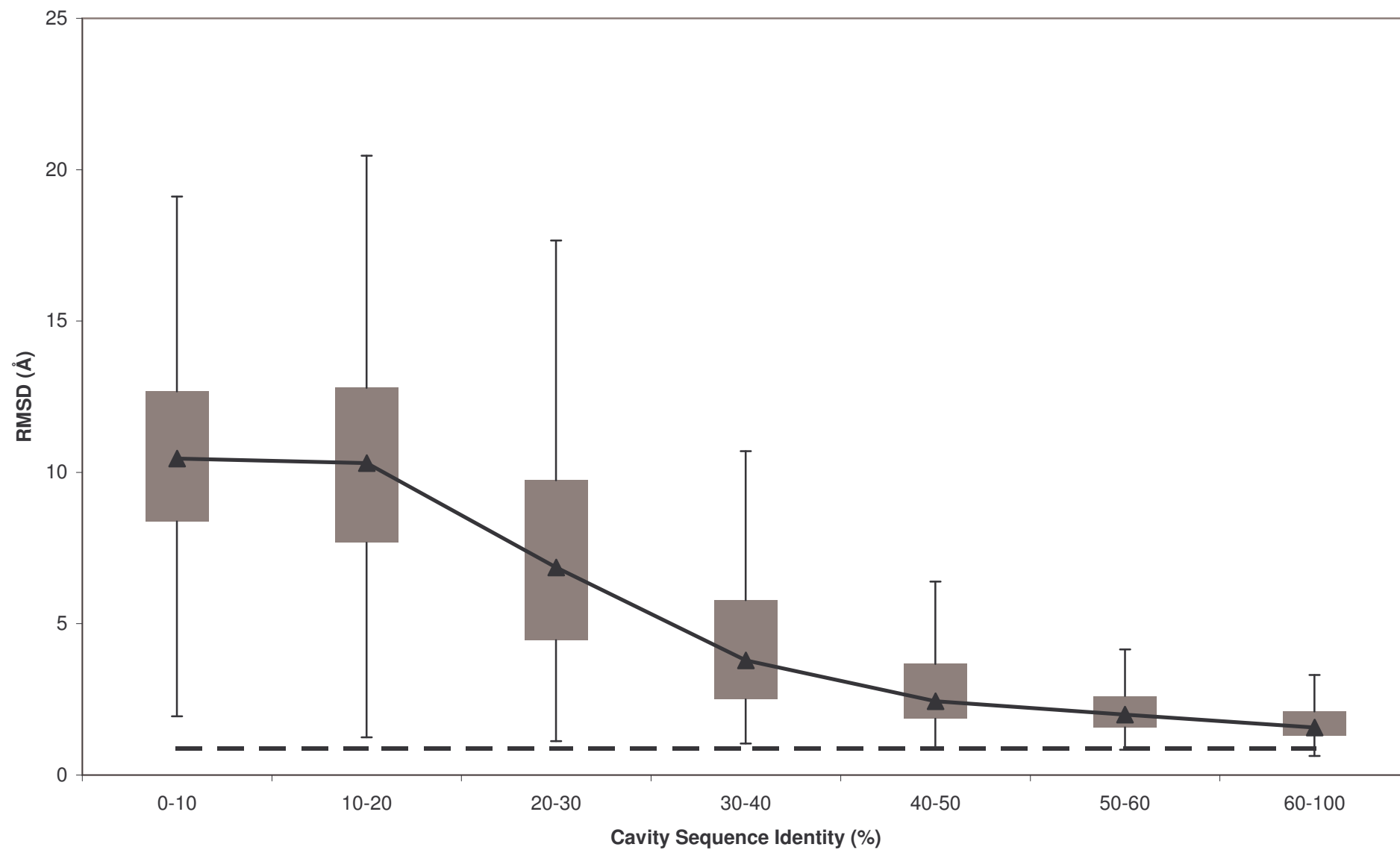

Supplement: Additional file 1 — RMSD vs. CLEFT SEQUENCE IDENTITY. The boxplot shows how cleft rmsd varies with target-template seq.id., with the latter computed for cleft residues only. The results are very similar to those shown in Figure 1 of the main body of the article, with rmsd improving as seq.id. approaches 100%; the quality transition observed below 20% is only slightly smoother. This result is in accordance with the study by DeWeese-Scott and Moult[34]. [file 1472-6807-8-2-S1.pdf]

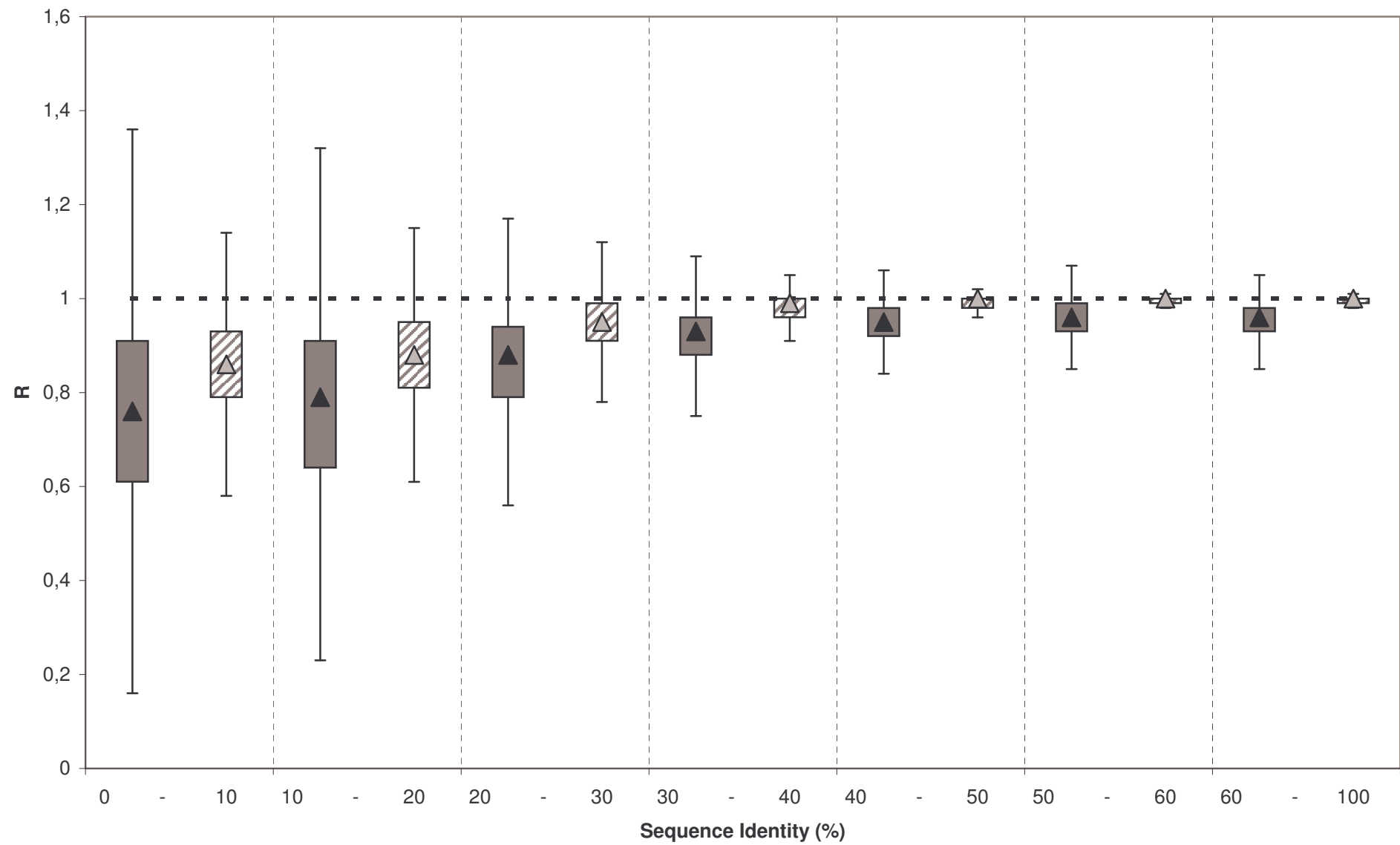

Supplement: Additional file 2 — SIDE-CHAIN CONTRIBUTION TO GDT_4 AND GDT_8 vs. SEQUENCE IDENTITY. To assess the contribution of side-chains to cleft models we computed R, the ratio between the percentage of side-chain atoms in the list of atoms contributing to a given GDT (GDT_1, GDT_2, etc) and the percentage of side-chain atoms in the cleft's set of contouring atoms. In the main body of the article we discuss the results for GDT_1 and GDT_2. In this figure we show the boxplot for GDT_4 and GDT_8. For GDT_4, which is associated with medium quality sub-structures, we observe that most of the models have R values below 1, thereby reflecting that main-chain atoms are modelled with better accuracy than side-chain atoms. For GDT_8, we observe that most R values are between 0.8 and 1, indicating that at this low quality level, almost all cavity atoms are included in the cleft model. Vertical dashed lines are used to separate the seq.id. bins. [file 1472-6807-8-2-S2.pdf]

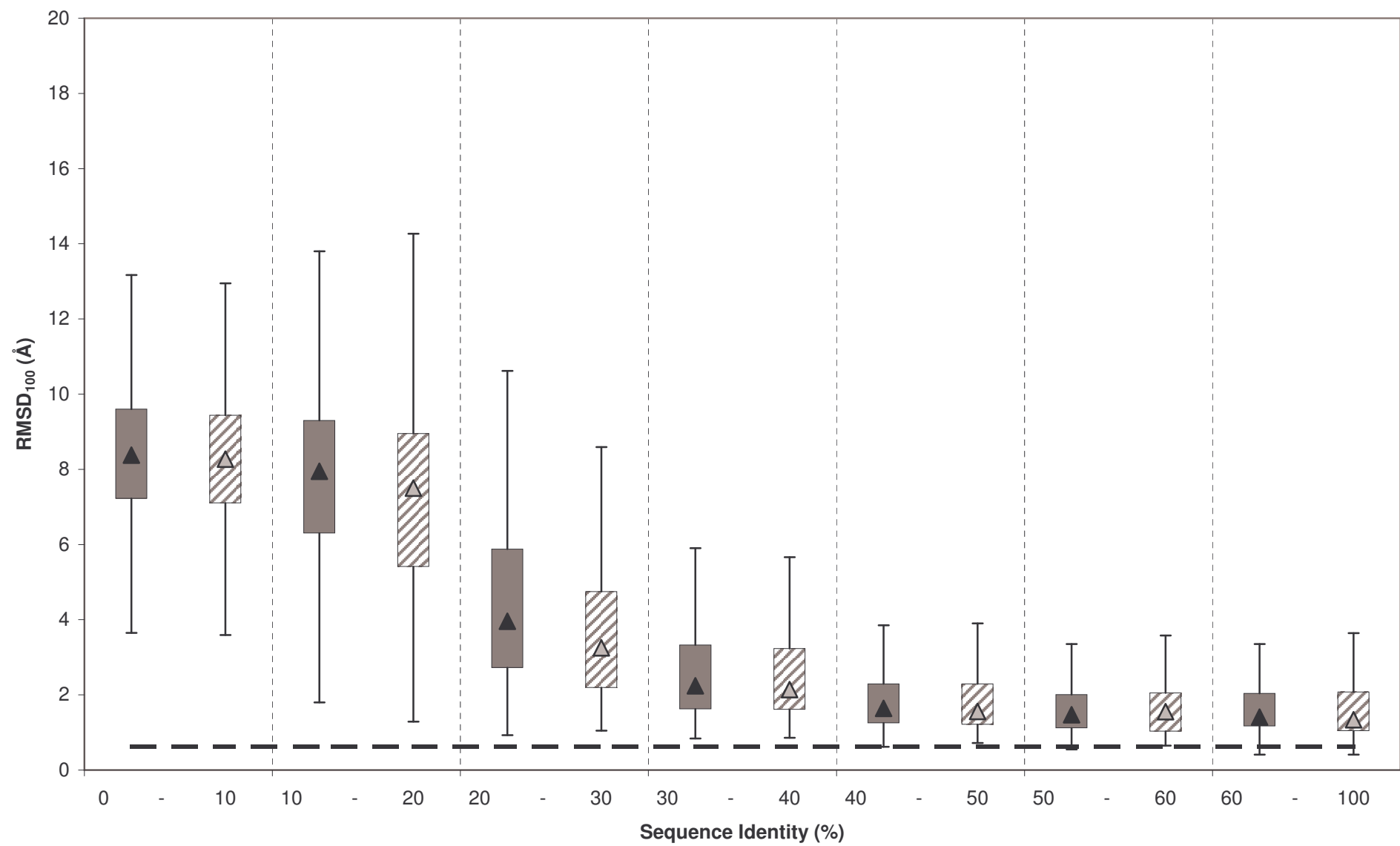

Supplement: Additional file 3 — Dependence of results on protein family. Several CATH[50] families are large and naturally contribute a larger number of models than smaller families to our results. To examine whether the latter show a specific behaviour, we reproduced the analysis of Figure 5 for families contributing less than 100 models each (dashed boxes). We subsequently compared the resulting rmsd100 distribution with that of the whole set of models (grey boxes). No substantial differences are observed between sets. Vertical dashed lines are used to separate the seq.id. bins. [file 1472-6807-8-2-S3.pdf]

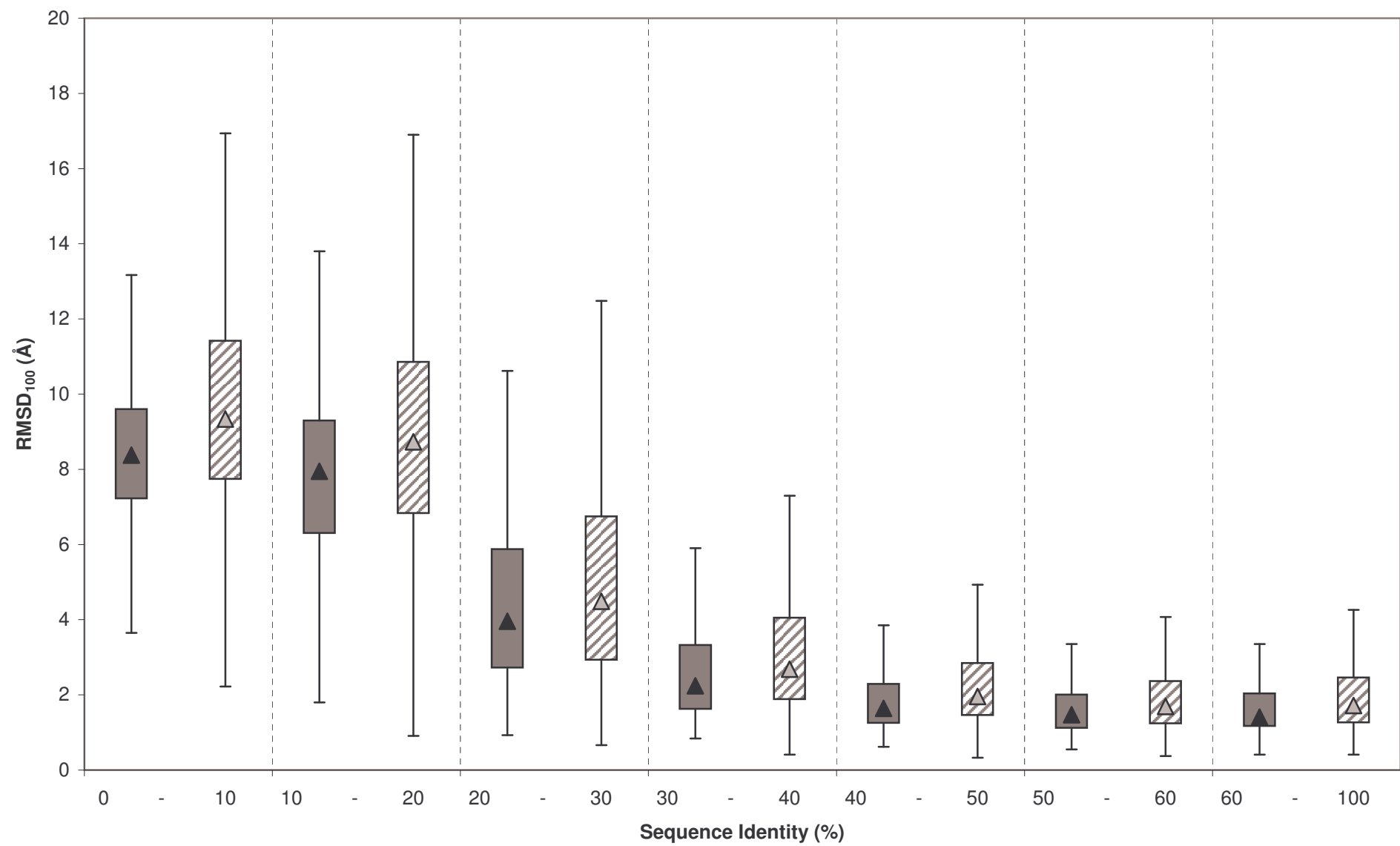

Supplement: Additional file 4 — Dependence of results on cavity rank. In some cases it may occur that the protein function locus is located in a secondary cleft rather than in the largest cavity. To explore the quality with which smaller clefts are modelled, we show the comparison between the rmsd100 distributions for the largest cleft (grey boxes) and the top five clefts (dashed boxes). The latter, particularly for seq.id. below 30%, tend to have poorer qualities thus suggesting that secondary clefts are reproduced with lower quality in comparative models. This poorer reproduction is probably because these cavities have a smaller number of matching residues in the target-template alignment. Vertical dashed lines are used to separate the seq.id. bins. [file 1472-6807-8-2-S4.pdf]
